# Incidence and type of complications in non-operated patients at a surgical ward

**DOI:** 10.1186/1754-9493-4-11

**Published:** 2010-07-20

**Authors:** Eelco J Veen, Maryska LG Janssen-Heijnen, Mariska AC de Jongh, Jan A Roukema

**Affiliations:** 1 Department of Surgery, Amphia hospital, Breda, the Netherlands; 2 Comprehensive Cancer Centre South, Eindhoven, the Netherlands; 3 Department of Surgery, St. Elisabeth Hospital, Tilburg, the Netherlands

## Abstract

**Background:**

This study was designed to analyze a group of non-operated patients admitted to our surgical ward for incidence and type of documented complication. We classified and categorised these complications according to the definition of the Association of Surgeons of the Netherlands (ASN). Our main interest was to identify adverse events for non-operated patients that are caused by medical management and thus preventable.

**Methods:**

Complications were prospectively collected in our registry, which is part of an electronic medical patient file, and in retrospective analysed. All non-operated patients admitted to our surgical ward between January 2003 and January 2006 have been analysed for type and incidence of complications.

**Results:**

We recorded 437 complications in 364 (8%) of 4602 non-operated patients and we categorised 196 (45%) of these events in the Hospital - Provider group. In this last category 161 (82%) events were related to medical management and appeared to be preventable. Numerous different types of complications were recorded (n = 69) among the 437 events. Of all the complications, 75 (17%) were found to be a negative effect/failure of therapy.

**Conclusion:**

The incidence of complications in non-operated patients at our surgical ward was 8%, with a great variety in types of events documented. Almost half of all complications (45%) were recorded in the Hospital-Provider category and appeared to be preventable, which needs further investigation.

## Introduction

Traditionally, surgical complications are recorded in relation to a procedure. A substantial rate of complications, though, is not associated with an operation. Non-operated patients admitted to a surgical ward have not been extensively analysed in literature, but the occurrence of complications leading to measurable disability appears to be related to medical management, as shown by in-hospital adverse events studies [1,2]. Others have shown that 53% of preventable complications are caused by problems in general ward management [3]. Many complications in non-operated patients are probably related to general ward management, which offers an opportunity for quality improvement. Nowadays, the discussion on safety and quality in health care argues for a much wider assessment of factors relevant for surgical outcome [4]. We have been documenting complications in our surgical department prospectively since 1986 [5]. Until 1995 we used to record only complications related to a surgical procedure, such as wound infection or post-operative haemorrhage. In our current evaluation of the whole surgical care process it is not the procedure, but the patient who is central, which is why we now also record complications in non-operated patients. In this paper we present an analysis of incidence and type of documented complications in a group of non-operated patients.

## Patients and Methods

### Patients

All patients who, in the period between January 2003 and January 2006, were admitted to our surgical department but not operated upon, were retrospectively analysed for incidence and type of complications. The setting was a non-university teaching hospital offering all major specialist services except cardio-surgery. The electronic medical file for all patients admitted to our surgical ward since 2003 contains the following data about patient, disease and treatment: "age, gender, ASA (*American Society of Anaesthesiologists Physical Status*) classification, hospital stay, ICD 10 Code, acute or elective procedure, and complications" [6]. The patient's diagnosis is filed into one the following categories: "General surgery, Traumatology, Vascular surgery, Gastro-intestinal surgery, and Oncology".

### Definition

To identify a complication we used the definition by the Association of Surgeons of the Netherlands (ASN): "Any state or event which is unfavourable to the patient's health, arises during admission or within 30 days after discharge, and either causes unintentional injury or requires additional treatment." Accordingly we restricted the follow-up period to 30 days after discharge. Over the years, this definition has expanded and now we also include undesirable events that have no directly noticeable negative effects on the patient's health or don't cause need for additional treatment. We distinguish two categories for these events, one category for negative effects caused by the intervention/drug/disease/organisation and another one for failures of therapy, as has been described by Clavien [7].

### Recording and coding of complications

Complications were prospectively recorded in our registry, which is part of the electronic medical patient file, and in retrospective analysed.

A complication was identified and recorded real time by one of the physicians of the surgical team (consisting of 10 surgical residents and 10 consultant surgeons). The electronic medical file can be accessed in every part of the hospital and the outpatient clinic, which makes recording easy. Both the in-hospital complications and those documented at the outpatient clinic were automatically presented at the daily surgical conference, where the surgical team discusses under which coding and type of complication they should be filed. Coding was done according to the classification systems of the Association of Surgeons of the Netherlands (ASN) and the Trauma Registry of the American College of Surgeons (TRACS) [8]. Neither system addresses the severity of complications. The system of the Association of Surgeons of the Netherlands uses four denominators to classify an event, including nature of the complication, anatomic localization, specification and additional description. The American College of Surgeons National Trauma Registry System was originally developed as a complication list to record the morbidity in trauma patient populations. The list explicitly defines complications and uses four-digit-codes. Although this list was developed for the trauma population, its design is broad and encompasses complications applicable to general surgery. Finally a free text description is recorded.

For simplicity's sake all complications documented in the non-operated group were divided into six categories, i.e. 1) Intervention/diagnostic - related events, 2) Infection-related, 3) Organ dysfunction (cardiopulmonary - renal - neurological - gastrointestinal), 4) Hospital - Provider related, 5) Drugs - related, 6) Miscellaneous.

The evaluator was the first author (EV), who was blinded for the complications documented in the registry and who was at the time not affiliated to the department. From his earlier working experience at the surgical department of the St Elisabeth hospital he was familiar with the registry and methods of registration. Additional events found by the evaluator were taken along in the total analysis. The follow-up period was chosen to be 30 days according to the definition and all patients were seen at the outpatient clinic within 30 days after discharge. The system was recently analysed by two independent reviewers for it's accuracy, a substantial interrater reliability was found, kappa score of 0,695.(unpublished data)

### Statistical analysis

Patient characteristics were compared using chi-square test and one-way ANOVA where appropriate. Comparison of length of stay was performed using the Wilcoxon rank-sum test because of the nonparametric distribution of this variable. Analyses were performed with the Statistical Package for the Social Sciences (SPSS 15.0 for Windows) software.

## Results

Between January 1, 2003, and January 1, 2006, 15866 patients were admitted at our surgical ward. In the period studied 4602 (29%) of all patients were not operated upon, and thus included in our study. Table 1 shows the number of non-operated patients admitted to our surgical ward and the number of complications documented per year. Three hundred sixty-four (8%) of 4602 patients of the study population had one or more complications. A total of 422 complications were recorded in these 364 patients, with 68 different types of events. Additionally, after evaluating the files of patients with complications (n = 364), 15 events were found and included in the analysis. There was a slight rise in the number of complications in the years 2004 and 2005 compared to 2003. However, characteristics of patients with complications did not reveal significant differences in age, gender and type of admission (acute/elective) over the years studied (see Table 2). Table 3 shows the characteristics of the six different complication categories. Almost half (n = 196; 45%) of all complications were categorised in the Hospital - Provider group. Most events were judged as factors related to medical management (n = 161; 82%), including diagnostic errors or delay's (n = 25; 13%), technical errors (n = 15; 8%), delay in MD response/obtaining consultation (n = 51; 26%), logistic problems, resulting, for instance, in delayed admission at the operation room, (n = 46; 9%) or incomplete hospital records (n = 24; 12%).

**Table 1:** The number of non-operated patients and registered complications admitted to the department of surgery at the St Elisabeth Hospital Tilburg

	Patients Admitted	Number of patients with complications		Number of Complications
***Year***	***N***	***N***	***%***	***N***
2003	1409	106	7,5%	125
2004	1613	127	7,9%	161
2005	1580	131	8,3%	151

Total	4602	364	8%	437

Fifteen complications found after evaluating all medical files have been included in this analysis.

**Table 2:** Characteristics of non-operated patients with complications admitted to the surgical ward of the St. Elisabeth hospital Tilburg

	2003 (n = 106)	2004 (n = 127)	2005 (n = 131)	Total n = 364	P-value
**Gender**					
Male	55 (52%)	70 (55%)	66 (51%)	190 (53%)	ns
Female	51 (48%)	57 (45%)	64 (49%)	172 (47%)	

**Age Mean (range)**	60 (0-94)	62 (2-92)	62 (2-94)	61 (0-94)	ns

**Hospital stay (days), median (interquartile range) †**	3 (2-7)	3 (2-7)	4 (2-9)	3 (2-8)	ns

**Admission**					ns
acute	45 (42%)	58 (46%)	45 (34%)	148 (40%)	
elective	61 (58%)	69 (54%)	86 (66%)	216 (60%)	

**† **Median (interquartile range)_Wilcoxon Signed Rank test

**Table 3:** Characteristics of non-operated patients according to the complication category between 2003 and 2006

	Intervention Diagnostic	Organ Dysfunction	Infection	Hospital - provider	Miscellaneous	Drug	P-value
**Number of complications**	N = 61 (14%)	N = 86 (20%)	N = 50 (11%)	N = 196 (45%)	N = 35 (8%)	N = 9 (2%)	
**(Total N = 437)**							

Nr of patients *	N = 59	N = 73	N = 45	N = 186	N = 35	N = 9	
***(Total N = 364)***							

**Gender:**							ns
*Male*	N = 25 (42%)	N = 37 (51%)	N = 22 (49%)	N = 105 (56%)	N = 19 (54%)	N = 5 (56%)	
*Female*	N = 34 (58%)	N = 36 (49%)	N = 23 (51%)	N = 81 (44%)	N = 16 (46%)	N = 4 (44%)	

**Age: †**	67.8 ± 12.6	70.7 ± 13.6	63.0 ± 15.7	58.4 ± 20.6	54.6 ± 17.9	69.8 ± 5.6	< 0.001

Diagnosis							< 0.001
(per patient)							
General	N = 2 (3%)	N = 13 (18%)	N = 11 (24%)	N = 47 (25%)	N = 12 (34%)	N = 3 (33%)	
Traumatology	N = 0 (0%)	N = 18 (25%)	N = 13 (29%)	N = 44 (24%)	N = 7 (20%)	N = 1 (11%)	
Vascular surgery	N = 52 (88%)	N = 29 (40%)	N = 6 (13%)	N = 61 (33%)	N = 6 (17%)	N = 4 (44%)	
Gastro-intestinal surgery	N = 4 (7%)	N = 10 (14%)	N = 14 (31%)	N = 15 (8%)	N = 5 (14%)	N = 0 (0%)	
Oncology	N = 1 (2%)	N = 3 (4%)	N = 1 (2%)	N = 19 (10%)	N = 5 (14%)	N = 1 (11%)	

**Hospital stay (days) ††**	4 (4-7)	8 (4-18)	9 (7-19)	2 (2-4)	2 (1-7)	10 (3-19)	< 0.001

* One patient could have complications in different categories.

**† **Mean ± standard deviation.

**†† **Median (interquartile range)_Wilcoxon Signed Rank test

In the organ dysfunction category, the patients' age was significantly higher compared to that of the other groups (P < 0.001), and 56 (65%) of the 85 complications recorded were cardiopulmonary related. In the Intervention/diagnostic (n = 61) category, significantly more patients were diagnosed with vascular pathology (see Table 3). Frequently recorded complications in this category include Bleeding/Arterial thrombosis/Dissection/Residual stenosis and pseudo-aneurysm formation (n = 44. 72%), which occurred in patients with peripheral arterial disease after treatment with Percutaneous Transluminal Angioplasty. Other events were related to colonoscopy, endoscopic retrograde pancreaticoduodenography or, for instance, iatrogenic pneumothorax after introducing a central venous catheter. The incidence of Infection - related complications in non-operated patients was 11% (n = 50). Most frequently documented infections were Urinary tract infection (n = 15. 30%), Pneumonia (n = 12. 24%), and Sepsis (n = 10. 20%). The category Drug related (n = 9. 0.2%) was small with 3 allergic reactions, 5 coagulopathy and one overdose of benzodiazepines. Six of those events rather were a negative effect of the drug. Of the 437 complications 75 (17%) actually were a negative effect/failure of therapy. Twenty-eight complications were found in the intervention/diagnostic group and were related to a dissection during an angioplastic procedure, resulting in the placement of a stent. Thirty-eight of the complications related to a negative effect/failure of therapy were found in the Miscellaneous (n = 26) and Hospital - Provider (n = 12) group, regarding patients who had their procedure postponed due to illness of the patient/inadequate regulation of preoperative measurements or logistic problems (for instance, no ICU or Operating Room capacity).

## Discussion

Quality assessment and improvement in surgery has traditionally concentrated on the registration of operation related complications. However, one third of all patients admitted to our surgical ward are not operated upon. A number of studies worldwide suggest that approximately 10% of patients admitted to the hospital suffer some kind of harm, about half of which could be prevented [9,10]. In a review on the incidence and nature of adverse events de Vries showed that a substantial part is surgically related, and at the same time varies considerably in incidence (3,2% to 16,6%) [10]. A review of the effects of study methodology on adverse outcome occurrence estimates that adverse outcomes occur in 16% (12-19%) of non-selected patients and in 18% (14-22%) of surgical patients [11]. None of these studies mention incidence of complications in non-operated surgical patients. Our study reveals that non-operated patients account for 8% of the incidence of complications. An earlier study by our group has shown an incidence of 27% in patients operated upon [5]. Comparing incidence with other studies remains difficult, especially as we use a broad definition of complications, resulting in as many as 65 different types of events recorded in this study. It has been suggested that differences in the interpretation of definitions could be more important than the difference in definition itself [11]. The broad definition used in our study has resulted in registration of 17% of all complications in the category negative effects and failure of therapy. However, these events are not complications from a traditional viewpoint. For instance, a dissection after an endovascular angioplasty needing stent placement was recorded, but inherent to the procedure. Recording and evaluating these types of events and their outcome will lead to a better understanding of the indication, patient population and treatment. Such information could be used in a local audit program for quality improvement. Our present study contains a group of patients with vascular pathology, in which a peripheral endovascular procedure or diagnostic angiography was performed. In our registry we do not record peripheral endovascular treatment as a surgical procedure, which could be discussed. This is due to the fact that the interventional radiology department, in cooperation with our vascular surgeons, performs these procedures. Data on documented complications for these interventions are diverse and not well defined in literature. Including patients with peripheral endovascular procedures and recording of negative effects or failure of therapy could be a flaw of this study. Almost half of all documented complications were recorded in the Hospital - Provider category. The events recorded in this category were diverse, related to medical management and probably preventable. Not all recorded events in this category cause injury or disability from a classical point of view [12,13]. For instance, lack of capacity at the Intensive Care Unit has been a challenge since our department became a Level 1 Trauma and referral centre for oncologic - head and neck/oesophagus - procedures. The resulting postponement of procedures was a major adverse event from the patient point of view. Events related to technical problems, such as wrongly placed intravascular devices/drain (thorax drainage), did not always result in an injury. Paying attention to and discussing adverse events, even if they did not cause injury or disability, makes everyone aware of potential major hazards.

Delay in diagnosis (n = 11) was documented 10 times in the traumatology population. The system provided a clear insight in the consequences of such events, which we analysed and used for improving quality in trauma care [14]. Delay in MD response was diverse, with 51 (26%) different types of events involving many aspects of care, from no culture swab performed (resulting in postponement of a procedure) to anticoagulants not started in a patient with pulmonary embolism. We did not explore the causes of our events, but according to the descriptions a substantial part may be attributed to mismanagement at our ward and therefore avoidable. These events provide an opportunity for further investment and improvement in the future. Therefore we recently started a study to analyse the causes of all documented adverse events in our clinic. Many studies have classified adverse events due to errors or negligence, and in this aspect preventable [13]. Analysing negligence and errors requires an estimation of error risk. Therefore epidemiological research is needed to determine standards of care, in which the entire process of care should be taken into account [15,16] Especially when case records are analysed in retrospective, the ubiquitous nature of hindsight bias should not be overlooked. Instead of focusing on the point where people went wrong, we should try to understand why their decisions and actions seemed to make sense at the time [17]. Errors will definitely be found but we should deal with these in an ethical way and we should exhaust our efforts in correcting the processes and situations that lead to it [18]. To analyze clinical incidents a broad framework has been developed of contributory factors that may affect practice and that include both error-producing conditions and latent failures. Emphasis should be put on identifying and rectifying "error-producing conditions" [19,20]. In this study we are not informed about the severity and consequences of the registered complications, which is a flaw of the study. Information about the impact of a complication on outcome is essential for further quality initiatives. In 2007 we adapted our registry to prospectively document possible consequences of complications.

## Conclusion

Thirty percent of the patients admitted to our surgical ward are not operated upon and in 8% of these cases complications were registered. Most complications recorded in non-operated patients were classified as Hospital - Provider events (n = 196) and 82% of those events were related to medical management. Various adverse events in this category appear to be preventable, which provides an opportunity for further investment and improvement. If we want to build a safer environment for the surgical patient, we should give the same priority to recording and analyzing complications in non-operated patients as to traditional procedure related complications.

## Competing interests

The authors declare that they have no competing interests.

## Authors' contributions

EV conceived of the study, analysed the data and wrote the first draft of the manuscript. MJ participated in the sequence alignment and drafting of the manuscript. MJ collected the data, assisted in data and statistical analysis. JR conceived of the study and participated in the design. All authors read and approved the final manuscript

## References

[B1] Leape LL Brennan TA Laird N Lawthers AG Localio AR Barnes BA Hebert L Newhouse JP Weiler PC Hiatt H The nature of adverse events in hospitalized patients. Results of the Harvard Medical Practice study 2 N Eng J Med 1991 324 377 84 10.1056/NEJM199102073240605 1824793

[B2] Vincent C Neale G Woloshynowych M Adverse events in British hospitals: preliminary retrospective record review BMJ 2001 322 517 19 10.1136/bmj.322.7285.517 11230064 PMC26554

[B3] Neale G Woloshynowich M Vincent C Exploring the causes of adverse events in NHS hospital practice J R Soc Med 2001 94 322 330 11418700 10.1177/014107680109400702 PMC1281594

[B4] Vincent C Moorthy K Sarker SK Chang A Darzi AW System Approaches to Surgical Quality and Safety: From Concept to Measurement Ann Surg 2004 239 475 482 10.1097/01.sla.0000118753.22830.41 15024308 PMC1356252

[B5] Veen E J Janssen-Heijnen M LG Leenen L PH Roukema J A The Registration of Complications in Surgery: A Learning Curve World J Surg 2005 402 409 10.1007/s00268-004-7358-8 15696399

[B6] Saklad M Grading of patients for surgical procedures Anesthesiology 1941 2 281 4 10.1097/00000542-194105000-00004

[B7] Clavien PA Sanabria JR Strasberg SM Proposed classification of complications of surgery with examples of utility in cholecystectomy Surgery 1992 111 5 518 26 1598671

[B8] American College of Surgeons Committee on Trauma Resources for the optimal care of the injured patient 1999 Chicago

[B9] Thomas EJ Brennan T Vincent CA Errors and adverse events in medicine: an overview Clinical Risk Management. Enhancing Patient Safety 2001 London: BM Publications 31 44

[B10] de Vries EN Ramrattan MA Smorenburg SM Gouma DJ Boermeester MA The incidence and nature of in-hospital adverse events: a systematic review Qual Saf health Care 2008 17 216 223 10.1136/qshc.2007.023622 18519629 PMC2569153

[B11] Marang-van de Mheen P J Hollander EJ F Job K Effects of study methodology on adverse outcome occurrence and mortality International Journal for Quality in Health Care 2007 19 399 406 10.1093/intqhc/mzm039 17875542

[B12] Brennan TA Leape LL Laird NM Hebert L Localio AR Lawthers AG Newhouse JP Weiler PC Hiatt HH Incidence of adverse events and negligence in hospitalized patients: results of the Harvard Medical Practice Study 1 N Engl J Med 1991 324 370 376 10.1056/NEJM199102073240604 1987460

[B13] Leape L L Reporting of adverse events N Engl J Med 2002 347 1633 1638 10.1056/NEJMNEJMhpr011493 12432059

[B14] Vles WJ Veen EJ Roukema JA Meeuwis JD Leenen LPH Consequences of delayed diagnosis in trauma patients: a prospective study J Am Coll Surg 2003 197 596 602 10.1016/S1072-7515(03)00601-X 14522329

[B15] Giard RWM The Epidemiology of medical errors: a few issues in methodology Ned Tijdschr Geneeskd 2005 149 2157 62 Dutch 16223075

[B16] Cuperus-Bosma JM Wagner C van der Wal G Patient safety in hospitals Ned Tijdschr Geneeskd 2005 149 2153 2156 Dutch 16223074

[B17] Henriksen K Kaplan H Hindsight bias, outcome knowledge and adaptive learning Qual Saf Health Care 2003 12 46 50 10.1136/qhc.12.suppl_2.ii46 PMC1765779 14645895

[B18] Krizek T J Surgical Error: Ethical issues of adverse events Arch Surg 2000 135 1359 1366 10.1001/archsurg.135.11.1359 11074896

[B19] Charles V Understanding and responding to adverse events N Engl J Med 2003 348 1051 1056 10.1056/NEJMhpr020760 12637617

[B20] Carter DC Adverse Events Br J Surg 2004 91 785 786 10.1002/bjs.4650 15227683

